# Adherence to Short-Duration Treatment (3HP) for Latent Tuberculosis among International Migrants in Manaus, Amazonas: Evaluation of the Efficacy of Different Treatment Modalities

**DOI:** 10.3390/microorganisms12081629

**Published:** 2024-08-09

**Authors:** Yan Mathias Alves, Thaís Zamboni Berra, Sonia Vivian de Jezus, Vânia Maria Silva Araújo, Jair dos Santos Pinheiro, Lara Bezerra de Oliveira de Assis, Marvis Canelonez, Daniel Souza Sacramento, Freddy Perez, Ethel Leonor Noia Maciel, Ricardo Alexandre Arcêncio

**Affiliations:** 1 Department of Maternal-Infant and Public Health Nursing, Ribeirão Preto College of Nursing, University of São Paulo, Ribeirão Preto 14040-902, Sao Paulo, Brazil; yan.alves@usp.br (Y.M.A.);; 2 Brazilian Tuberculosis Research Network (REDE-TB), Rio de Janeiro 21941-904, Rio de Janeiro, Brazil; 3 Sinop Campus, Federal University of Mato Grosso (UFMT), Sinop 78550-728, Mato Grosso, Brazil; 4 State Tuberculosis Control Program of Amazonas, Manaus 69093-018, Amazonas, Brazil; jpsantos.jair@gmail.com (J.d.S.P.); larabezerradeassis@gmail.com (L.B.d.O.d.A.);; 5 Municipal Tuberculosis Control Program, Manaus 69049-110, Amazonas, Brazil; 6 Communicable Diseases Prevention, Control, and Elimination and Environmental Determinants of Health Department, Pan American Health Organization, Washington, DC 20037, USA; perezf@paho.org; 7 Department of Health Sciences, Federal University of Health Sciences of Porto Alegre, Porto Alegre 90050-170, Rio Grande do Sul, Brazil; 8 Ministry of Health, Secretariat for Health Surveillance and the Environment, Brasília 70058-900, Distrito Federal, Brazil

**Keywords:** tuberculosis, migrants, therapy

## Abstract

Migration, a multifaceted phenomenon, has a significant impact on health. Migrants perform similar movement patterns within their country of origin, in transit, and in the country of destination, thus making it difficult to monitor TB treatment throughout the journey. The objective was to compare the effectiveness of different treatment modalities in adherence to the short-term regimen for LTBI (3HP) among international migrants and refugees. This is a quasi-experimental study conducted in Manaus-AM. The study population was made up of international migrants. The certification and monitoring of medication intake employed three strategies: self-administration (SA), directly observed conventional therapy (DOT), and Video Telemonitoring System for Tuberculosis Treatment (VDOT). The VDOT group and SA group exhibited the lowest rate of treatment dropout or interruption at 16.1%, followed by the DOT group at 23.1%. The results suggest that the most effective strategy for ensuring adherence among migrants and refugees was VDOT (OR_adj 0.26; CI 0.7–0.94), suggesting that migrants may be more likely to adhere to and complete their treatment. The results show that relying on different treatment strategies, adapted to the individuals’ needs and risk factors, is a viable and effective way of providing person-centered TB care.

## 1. Introduction

Tuberculosis (TB) is one of the ten leading causes of death globally and is intricately linked to poverty, social exclusion, unequal income distribution, and widespread geographic dispersion [1]. As part of the End TB Strategy, global representatives met in the United Nations in New York, reiterating the commitment to end the TB epidemic by 2030 and acknowledging the importance of prioritizing at-risk groups and vulnerable individuals, particularly international migrants and refugees [2].

An estimated one-fourth of the global population is estimated to have been infected with *Mycobacterium tuberculosis* without the presence of clinical signs [3], carrying a 5–10% risk of progressing to active TB. These individuals are defined as having latent tuberculosis infection (LTBI), making the treatment of LTBI crucial to interrupt the transmission chain and the development of TB disease [4]. In 2022, an estimated 10.6 million people developed active TB, resulting in 1.3 million deaths worldwide. Brazil, recording 78 thousand cases in 2022, stands as the country with the highest TB burden in the Americas [5,6,7].

LTBI is a condition in which a person is infected with the TB bacillus but does not show signs or symptoms of active disease and is not contagious. However, people with LTBI have an increased risk of developing active TB, as the bacillus can be reactivated if the immune response is altered or compromised [8,9]. For the World Health Organization (WHO), TB continues to be the main cause of death from an infectious agent in the world, with Brazil classified among the 30 countries with the highest TB burden in the world, with an incidence of 36.3 cases per 100,000 inhabitants and a mortality rate of 2.3 deaths per 100,000 inhabitants in 2022 [10]. Particularly amid the health crisis induced by the COVID-19 pandemic, there is a pressing need for innovative approaches to improve and optimize therapeutic interventions [11]. According to estimates from the Stop TB Partnership, the COVID-19 pandemic could lead to an increase of 6.3 million cases and 1.4 million TB-related deaths between 2020 and 2025, considering the 30 countries with the highest burden of the disease [12].

According to the WHO, the systematic testing and treatment of LTBI should be carried out in people living with HIV, adults and children who are contacts of pulmonary TB cases, patients starting treatment with anti-tumor necrosis factor (TNF), patients on dialysis, patients preparing for organ or hematological transplants, patients with silicosis, prisoners, healthcare professionals, immigrants from countries with a high TB burden, homeless people, and illicit drug users. Either interferon-gamma release assays (IGRAs) or the Mantoux tuberculin skin test (TST) should be used to test for LTBI [13]. The tuberculin skin test (PT) should be interpreted as suggestive of M. tuberculosis infection, regardless of the BCG vaccination rate. It should be considered positive when ≥5 mm and negative when <5 mm, indicating treatment for LTBI [14,15].

One of the main barriers to TB treatment adherence and LTBI is its prolonged duration [16]. In response, the WHO endorsed a novel approach for the treatment of LTBI, the 3HP regimen, which combines high doses of isoniazid (H) and rifapentine (P) administered once weekly for three months, optionally accompanied by directly observed therapy (DOT) or self-administration (SA). This regimen has been incorporated by the Brazilian Ministry of Health [17,18]. The Unified Health System (SUS) currently offers three therapeutic regimens for LTBI: isoniazid, rifampicin, and 3HP. The first requires nine months of treatment (270 daily doses), while the second takes four months (120 doses). Rifapentine associated with isoniazid is administered for three months (12 weekly doses). This significant reduction in doses ensures better adherence, with fewer adverse effects, contributing to the effectiveness of the treatment [19].

In addressing the healthcare gaps exacerbated by the COVID-19 pandemic, the WHO established a Global Task Force on Digital Health for TB to support the development of health innovations for TB prevention, treatment, and transmission interruption. In 2017, video-observed treatment (VOT) was endorsed as an alternative to traditional DOT, enabling remote treatment monitoring whenever feasible [20].

Global migration, a multifaceted phenomenon, has a significant impact on health due to exposures along migratory routes and epidemiological disparities in migrants’ countries of origin and destination [21]. An estimated 272 million individuals currently reside outside their home countries [21].

Migrants perform similar movement patterns inside their home country, in transit, and in the country of destination, thus hindering the follow-up of their TB treatment throughout their journey [22]. The COVID-19 pandemic has worsened the monitoring of treatment for this population due to the social distancing measures imposed in the country by the Brazilian Ministry of Health.

In response to the pandemic, the Video Telemonitoring System for Tuberculosis Treatment (VDOT), an app used to monitor TB treatment, was piloted in Ribeirão Preto, São Paulo, Brazil, showing promising results [23]. According to Story et al. (2016) [24], VOT surpasses DOT by enabling TB patients to use their smartphones to record medication intake videos, thereby eliminating distance barriers, reducing travel costs to healthcare facilities, and empowering individuals to choose when and where to go for medication. The study further highlights the enhanced efficiency of health services in monitoring patients with VOT.

VOT has proven effective across various populations, leading to reduced treatment dropout rates, lower costs, and a better acceptance of observed treatment (96% adherence with VOT compared to 46% for DOT). Improved adherence enhances treatment outcomes, decreases retreatment rates, and mitigates drug resistance development [25].

Given the aforementioned context and the implementation of short-course preventive treatment among migrants with LTBI, this study aimed to compare the efficacy of different treatment modalities (SA, DOT, or VDOT) in adhering to the short-course regimen for LTBI (3HP) among international migrants and refugees in Manaus-AM, Brazil.

The study is pertinent as it suggests that the short-course regimen may facilitate treatment adherence, particularly among international migrants who undergo constant migration within Brazil. Moreover, the VDOT tool promises greater treatment oversight, even if participants relocate to other cities, thereby contributing to interrupting the transmission cycle and preventing active TB development.

## 2. Materials and Methods

**Figure 1:**
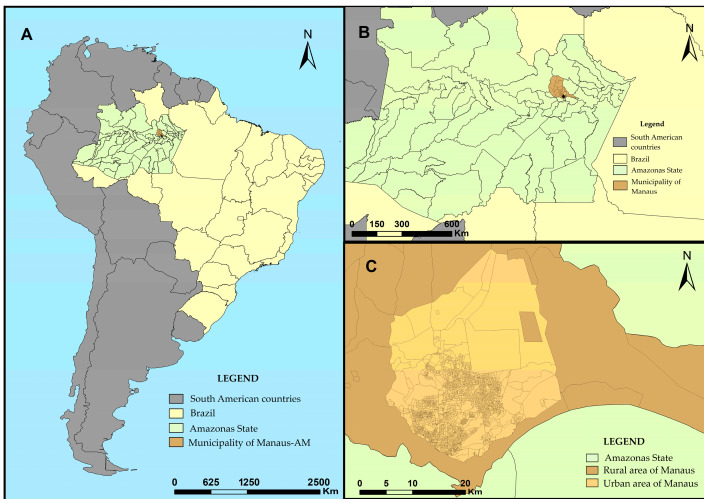
Geographic location of the municipality of Manaus, Amazonas, Brazil. Source: the authors. Study location and rationale. (**A**) South American Countries, (**B**) Amazonas State, (**C**) Munipality of Manaus.

This is a quasi-experimental study conducted in Manaus, the capital of Amazonas state in northern Brazil. With a population of 2.21 million, Manaus ranks among Brazil’s largest cities and serves as the primary economic center for the region, hosting various major industries within the Manaus Free Zone (ZFM) [26]. The municipality comprises 63 districts across five administrative areas: north, south, east, central-west, and central-south. Manaus is also divided into 2461 census tracts, 2420 of which are urban and are part of this study [27] (Figure 1).

Manaus was selected as the study site due to its significant burden of TB, ranking among the cities with the highest number of TB cases in Brazil. The city experiences a intense migratory flow, particularly from Venezuela, and serves as the destination for the Warao indigenous group. Manaus, as the state capital with the highest TB incidence in Brazil, is considered hyperendemic for TB. According to United Nations data published in June 2021, of the currently estimated 5.4 million Venezuelan refugees in the world, 260 thousand are living in Brazil, at least 20 thousand of whom in Manaus [28].

According to Brazil’s Information System on Diseases of Notification (SINAN), the municipality recorded 2667 new TB cases in 2022, with an incidence of 118.2 cases per 100 thousand inhabitants, and 1021 individuals in treatment for latent TB infection (LTBI) in 2022 [29]. These figures surpass the national TB incidence rate of 36.3 TB cases/100,000 inhabitants in Brazil [30]. According to the WHO, the estimated national tuberculosis incidence rate in Venezuela in 2022 was 46 cases per 100,000 inhabitants [31]. In view of this, it is clear that the city of Manaus is an endemic location for TB, since its TB incidence rate is significantly above the national average. The comparison with the TB incidence rate in Venezuela, which is also higher than the Brazilian average, further reinforces the characterization of Manaus-AM, Brazil, and Venezuela as endemic regions for the disease.

### 2.1. Study Population

The study population comprised international migrants diagnosed with LTBI based on tuberculin skin test (PT/PPD) induration (>5 mm) observed 72 h after intradermal application to the forearm performed by a qualified healthcare professional between December 2022 and October 2023.

### 2.2. Procedures

LTBI treatment was carried out in international migrants identified through active case finding. The migrants were identified with the assistance of non-governmental organizations (NGOs) and religious pastors that support shelters in communities/slums in Manaus-AM. The project also included the identification of participants in municipal and state shelters and accommodation of the Municipal Health Department of Manaus (SEMSA). The treatment of these patients followed the treatment protocol recently approved by the Brazilian Ministry of Health. Participants received rifapentine in combination with isoniazid in weekly doses for three months, totaling 12 consecutive weekly doses.

Drug intake certification and monitoring employed three strategies: self-administration (SA), conventional directly observed therapy (DOT), or the use of the VDOT app (Video Telemonitoring System for Tuberculosis Treatment). This approach accommodated the recent recommendation by the U.S. Centers for Disease Control and Prevention (CDC-USA) supporting the self-administration of 3HP for individuals ≥ 2 years of age (under the supervision of parents or guardians).

Importantly, LTBI treatment in migrants using the 3HP regimen is not routine in health services, nor is the use of the VDOT app. Hence, this study complements and enhances existing activities conducted by the Manaus municipal health services.

The tuberculin skin test was administered by trained health workers from the Manaus municipal health service using the purified protein derivative test (PPD RT23), in a dose equivalent to 2 units of tuberculin. Test reading was performed with a millimetric ruler by the same health worker who applied the PPD.

Inclusion criteria encompassed international migrants in Manaus, regardless of their home country or legal status in Brazil, who understood Portuguese, Spanish, or Warao. Participants were required to consent to undergo the PPD test, and with a positive test (>5 mm), to undergo chest X-ray imaging, return for test reading (48 to 72 h after inoculation), and initiate treatment for LTBI with the 3HP regimen.

The exclusion criteria included patients already undergoing treatment for active TB, having any immunosuppressive disease (such as HIV or others), presenting a negative PPD result (<5 mm), failing to return for test reading within 72 h, presenting chest X-ray findings indicative of active TB, declining treatment for LTBI with the 3HP regimen, or refusing monitoring treatment according to the allocated modality (SA, conventional DOT, or VDOT). Furthermore, women who were breastfeeding at the time of participant screening and people under 18 years old were also excluded. 

It is important to mention that after completing treatment, PPD does not convert to negative. According to guidelines from the Ministry of Health [32], in patients with a history of PT ≥ 5mm or reactive IGRA who have already received treatment for LTBI or have undergone treatment for active TB previously, at any time in life, it is not recommended to treat LTBI again, except when they have been exposed again to a risk of *M. tuberculosis*.

They were monitored via SA, DOT, or VDOT, using adapted arrangements, as described below.

### 2.3. Facilitated Self-Administered Treatment Group (SA)

Participants assigned to self-administered treatment followed the 3HP regimen once a week for 12 weeks. They received a medication card to record their weekly doses, were instructed to contact the study’s reference health service in case of side effects or complications, and received a telephone number for information and scheduling. Participants were requested to present their medication card during follow-up visits.

### 2.4. Directly Observed Treatment Group (DOT)

International migrants following conventional DOT adhered to the 3HP regimen once a week for 12 weeks under direct observation. Medication intake occurred at health services, the patient’s residence, or other locations designated by the health workers or the participant.

### 2.5. Video Directly Observed Treatment Group (VDOT)

Participants assigned to VDOT followed the 3HP regimen once a week for 12 weeks, recording medication intake via a smartphone app. Participants received instructions on app usage and were given a demonstration at the health unit. They recorded their medication intake using the app, which was then validated by the health team. Participants were equipped with 4G internet packages if needed. A designated administrator managed the app, overseeing participant adherence and facilitating communication between participants and health workers.

The health workers had access to the multidisciplinary notebook in the VDOT app, where they managed the interactive medication schedule. They used the technology to send optimistic messages to encourage the continuation of treatment.

Regardless of the assigned treatment modality, all participants were followed for 12 weeks, with treatment considered complete once all the scheduled medications had been taken. Participants that failed to complete the treatment were classified as “treatment incomplete”.

The way participants were allocated to monitoring groups (SA, DOT, or VDOT) was carried out randomly so that there would be no interference and participants had the same chance of being allocated to any of the groups. Participant information was added to the REDCap platform and through a functionality of the application participants were randomly distributed among the three arms of the study. Additionally, researchers were unable to change the allocation of any study participants.

### 2.6. Statistical Analysis

Initially, after the validation and standardization of the database, a descriptive analysis of the variables referring to the participants’ characteristics (sex, race/color/ethnicity, and marital status) was carried out, with a calculation of absolute and relative frequencies, with the aim of understanding the population of international migrants identified with LTBI in Manaus, and grouped according to the treatment modality to which the study participant was allocated.

With the aim of comparing the efficacy of the different treatments analyzed to verify adherence to the short-course regimen for LTBI (3HP) among international migrants and refugees, a logistic regression model was initially constructed in which the independent variables considered were the treatment modalities, while adherence to treatment (completed treatment: yes—‘0’ or no—‘1’) served as the dependent variable. The odds ratio (OR) and its respective 95% confidence intervals (CI95%) were calculated. 

Fit measures, including 2 Log likelihood, Cox–Snell R^2^, and Nagelkerke R^2^, were calculated to assess the performance of the logistic regression model, serving as indicators of the overall fit of the model to the data. The significance of each coefficient estimate was assessed using the Wald test, which determines whether the coefficient for each predictor variable differs significantly from zero. A significance level of α = 0.05 was used to determine statistical significance.

Finally, for those international migrants who did not complete the treatment for LTBI, a descriptive analysis was carried out on the variables referring to the characteristics of the participants (sex, race/color/ethnicity, and marital status) referring to these cases, with a calculation of absolute and relative frequencies, with the aim of knowing the population of international migrants identified with LTBI in Manaus who did not complete the treatment. All analyses were conducted using IBM SPSS Statistics version 22 software.

### 2.7. Ethical Approval

This study adhered to the guidelines outlined in Resolution 466 of 2012 and Resolution 510 of 2016 by the Brazilian National Council on Research Ethics (CONEP). Approval for this study was obtained from the Institutional Review Board of the Federal University of Espírito Santo (UFES) under review number CAEE 49633221.1.0000.5060, CONEP, as well as from the Pan American Health Organization Ethics Review Committee (PAHOERC), under review number 0484.02. All participants provided informed consent, which was accessible in online format. Consent was recorded online, and a copy was subsequently sent to each participant’s e-mail address.

## 3. Results

A total of 259 migrants were identified by the study team. During the initial screening stage for LTBI, all participants underwent tuberculin skin testing. Subsequently, 28 participants failed to return for test reading within the designated timeframe (48 to 72 h post application). Among the remaining 231 individuals who returned for test reading, 116 tested PPD-negative (<5 mm), while 115 tested positive for LTBI.

Participants testing negative for LTBI (<5 mm) were excluded from the study as they did not meet the inclusion criterion of positive PPD. Those testing positive (≥5 mm) continued in the study and underwent a chest X-ray, followed by medical evaluation to confirm study eligibility. Out of the 115 participants referred for chest X-ray, 112 completed the test, while 3 participants were excluded for failure to attend the scheduled appointment. 

**Figure 2:**
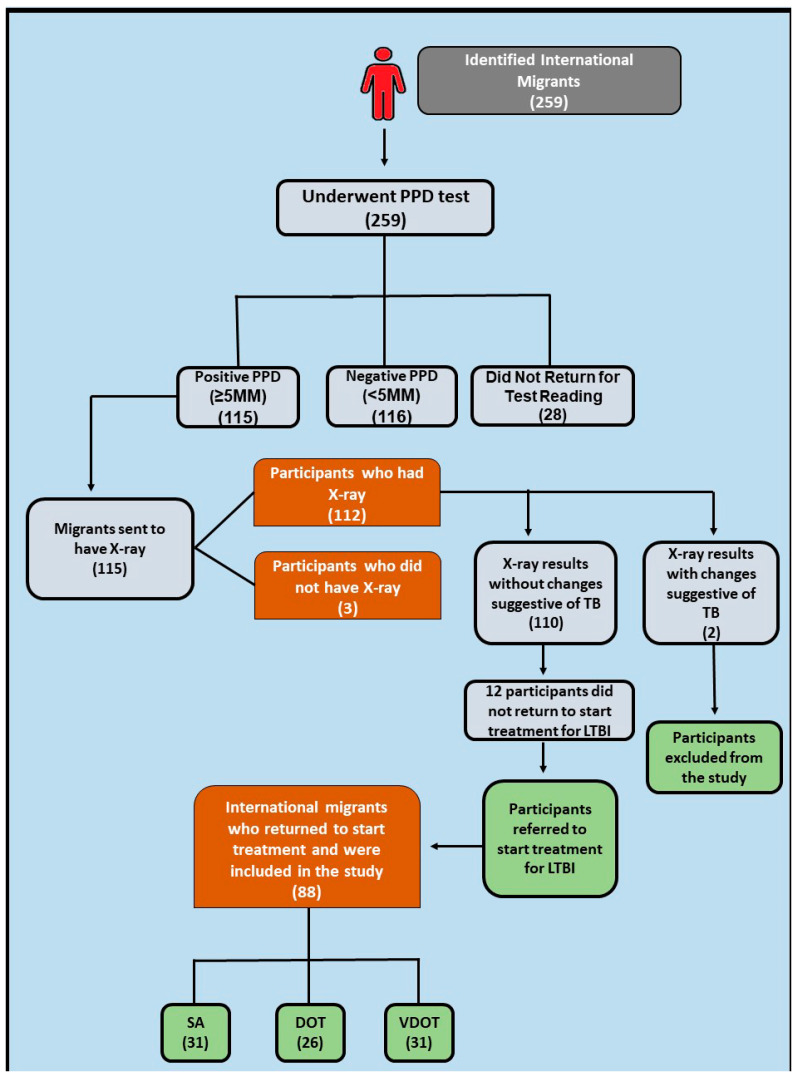
Flowchart with the study stages.

Among the 112 individuals who underwent a chest X-ray, 2 showed findings suggestive of TB. These individuals were referred to a medical specialist within the Manaus health services network for further evaluation. Following assessment by a physician contracted by the project, in accordance with the study criteria, these two individuals were excluded from the study. In addition, 12 participants did not return after taking the X-ray to start treatment for LTBI. Consequently, the final cohort compromised 88 international migrants with LTBI. Figure 2 provides the flowchart of the study’s stages and the number of participants included in each stage.

Participants were assigned to the SA (31), DOT (26), or VDOT (31) groups. Table 1 illustrates the distribution of gender across the three groups, revealing a prominent male representation with 58.1% (*n*: 18) in the SA group, 57.5% (*n*: 15) in the DOT group, and 51.6% (*n*: 16) in the VDOT group. The sample showed diversity in race/color/ethnicity; however, individuals self-identifying as brown constituting the majority across all three groups. As for marital status, the majority of participants in each group were single. The table also presents the treatment outcomes categorized by treatment modality. The VDOT group and SA group exhibited the lowest rate of treatment dropout or interruption at 16.1%, follow by the DOT group at 23.1%. 

Table 2 illustrate the gender distribution among the three groups according to treatment completion and incompleteness, revealing a prominent male representation of completing and not completing treatment. Regarding race/color/ethnicity, with individuals who self-identified as brown constituting the majority who completed treatment, among those who did not complete treatment, the majority self-identified as indigenous. Regarding marital status, the majority of both groups were single.

In Table 3, the efficacy of each treatment modality in promoting adherence to the short-course treatment (3HP regimen) is presented. The results suggest that the most effective strategy for ensuring adherence among migrants and refugees was VDOT (OR_adj: 0.26; CI: 0.7–0.94), suggesting that migrants may be more likely to adhere to and complete their treatment, thus demonstrating the efficacy of this approach. Conversely, individuals under SA are at a higher risk of not completing their treatment (OR_adj: 9.93; CI: 1.96–50.30). Regarding traditional DOT (OR_adj: 0.59; CI: 0.14–2.38), there is no evidence suggesting its contribution to adherence or default. The Cox–Snell R^2^, ranging from 0 to 1, with values closer to 1 indicating a better model fit, was found to be 0.32 in our study. Additionally, the Nagelkerke R^2^ was 0.43, suggesting that the model explains 32% of the variability in the efficacy of adherence to TB preventive treatment among migrants and refugees.

Among the participants who did not complete treatment with the 3HP regimen, the majority were males (70.59%), indigenous (47.06%), and single (70.59%). The mean age was 35 years, ranging from 19 to 58 years, with a standard deviation of 13.68 years. The TST/PPD results revealed a mean induration size of 11.5 mm, with a minimum of 5 mm and maximum of 21 mm, and a SD of 4.27 (Table 4).

## 4. Discussion

The objective was to compare the efficacy of different treatment modalities (SA, DOT, and VDOT) in adherence to the short-course regimen for LTBI (3HP) among international migrants. Of the 259 international migrants, the majority were female (56.0%), predominantly single (69.1%), and with ethnic diversity, with a predominance of brown individuals (55.6%). The participants’ mean age was 37.8 years. The most effective strategy for ensuring adherence among migrants and refugees was VDOT (OR_adj 0.26; CI 0.7–0.94), and we verified that individuals under SA are at a higher risk of not completing their treatment (OR_adj 9.93; CI 1.96–50.30).

The presence of international migrants in Brazil grew exponentially from 2011 to 2020, reflecting intrinsic vulnerabilities that can lead to inequalities in healthcare access. Barriers to healthcare access for international migrants include cultural differences, low socioeconomic status, language difficulties, a lack of documentation, alack of medical history, social isolation, and discrimination (mainly racism and xenophobia) [33]. Health teams also face challenges in providing care to this population, including language barriers and difficulty locating individuals due to frequent changes in residence or a fear of disclosing their address, particularly among refugees [33].

TB and LTBI are closely linked to living conditions such as access to basic sanitation, social determinants of health, and access to healthcare. The prevalence of cases among migrants can be attributed to conditions in their home country and during transit where risks to physical, mental, and social wellbeing are prevalent. Additionally, the migrants’ health status prior to departure from their place of origin and access to healthcare prior to migration play a significant role [34].

The known risk factors and social determinants of TB vary before, during, and after migration. TB burden in the home country, influenced by socioeconomic factors and healthcare capacity, contributes significantly [35].

Some countries with low TB incidence reported an increase in TB rates due to recent increases in the number of asylum-seekers and other migrants from countries where TB is endemic. Various TB prevention strategies targeted to populations born abroad can also impact TB rates and explain some variations. For example, countries have different TB screening policies for migrants. For more than 20 years, Australia, Canada, New Zealand, and the United States have required pre-migration screening for active TB in visa applicants. Evaluations in the United States have shown that the increase in TB detection prior to migration correlates with reduced notification rates in the first years after arrival. This approach thus appears effective for detecting prevalent active TB prior to migration and reducing TB notification after arrival in the host country [35].

International migrants face a particularly high risk of acquiring TB due to hazardous migration conditions. For example, a recent study of asylum-seekers in Switzerland reported an association between land and maritime transit and an increased risk of LTBI. The resulting high risk of TB infection or TB disease may be related to the transit conditions or the underlying social and economic conditions before and during migration [34].

The WHO emphasizes the importance of a free TB diagnosis and the treatment of LTBI for all international migrants to maximize healthcare access. Countries should guarantee easy access to low-cost installations where undocumented migrants can receive care without fear of being reported to immigration authorities, besides guaranteeing that undocumented international migrants diagnosed with active TB are not deported during their treatment [34].

Recent trends indicate a feminization of migration, with more women and children migrating internationally [36]. This social phenomenon began in 2015 and has been consolidated with the growing number of women crossing international borders, alongside the increase in the number of women in the general population. There has also been a new process in international migrations to Brazil, with the arrival of more children and adolescents [37]. Tonhati and Pereda (2021) [38] also emphasize the importance of Venezuelan female immigrants in intensifying this process of feminization. Besides increasing the number of female immigrants in the country, they have also contributed to the rise in their participation in the formal labor market. Given the significant role these women play in the current migration scenario in Brazil, there is a need to direct a focused and particular attention towards them [39]. 

A recurring characteristic of these women is that they often seek healthcare not for their own health, but to ensure the health of their children and families. It is evident that the condition of being a woman, both in family and social relationships, leads to a specific role that is repeated in their experiences, regardless of their nationalities. In sum, it can be said that migrant women reach an even more critical degree of social vulnerability, as they carry not only the condition of being a migrant but also a social burden related to gender issues. This affects their relationships, work, health, and survival conditions [39]. Given this, the higher number of women in this study can be understood as a reflection of their role in caring for the health of their family members, prompting them to seek more access to healthcare services and to be more concerned with their family’s health.

Women’s health literacy levels and their communication with healthcare providers might differ from men’s, potentially affecting their understanding of the importance of adherence to treatment regimens like 3HP for LTBI. Women might access healthcare services differently, possibly attending clinics for maternal and child health services, which could be leveraged to improve adherence to LTBI treatment by integrating TB care with other healthcare services they are already utilizing.

Despite the recognized vulnerability of international migrants to TB, few studies have addressed the prevalence of TB and/or the risk of LTBI in this population, particularly concerning short-duration preventive treatment. The study demonstrated a high number of positive PPD, which deserves attention from local health authorities, revealing a prevalence of LTBI among international migrants living in Manaus, with approximately 49.8% of PPD read showing positive results. Considering that some participants did not return for the reading, this number could be higher, thus raising an alarm. 

Manaus-AM recorded an incidence of TB of 118.2 cases per 100 thousand inhabitants in 2022 [29]. According to the WHO, the estimated national incidence rate of tuberculosis in Venezuela in 2022 was 46 cases per 100,000 inhabitants [31], far from targets aligned with commitments international goals such as the 2030 Agenda for Sustainable Development and aims to reduce the incidence of TB to fewer than 10 cases per 100 thousand inhabitants and fewer than 230 deaths by 2035 [40].

The participants received different treatment strategies, but all followed the 3HP regimen recommended by the CDC-USA for the treatment of TB infection [41]. A significant portion of the participants were indigenous Venezuelans, the Warao people originally from the Orinoco Delta in the state of Delta Amacuro, highlighting the diversity within the migrant population. 

The Warao people do not travel alone. Both the kinship networks and the groups formed along the migratory routes are crucially important for the strategies of mobility and settlement as the indigenous population moves in search of sustainability in a new context. It is important to consider the Warao people’s mobility as a cultural characteristic and a social and economic strategy that promotes the circulation of both merchandise and especially fundamental personal relations in the definition of social and political roles (for example, the affirmation and constitution of kinship and leadership) [42].

Brazil’s legislation is consistent with recommendations by international agencies on access to health for immigrants and refugees. Law 8.080/1990 regulates the Unified Health System (SUS), and Law 13.445/2018 regulates migration in the country, guaranteeing the right to health for immigrants in Brazil’s territory, regardless of their migratory status. Brazil is the only country in South America with a free and universal public health system, so many international migrants enter the country to obtain access to health treatments [33]. However, there is no specific public policy for TB control in the international migrant population.

DOT or supervised treatment is used to support and monitor the treatment of individuals with TB, consisting of a health worker’s observation of the patient’s TB drug intake, preferably every weekday. This method allows interaction and the establishment of a bond with the patient and requires committed and humanized care by health workers [43]. Scheduling for the health worker to watch the medication being taken can interfere with the patient’s job, schooling, and other daily activities, and it may be difficult to organize the health worker’s transportation to conduct DOT. With community-based DOT, the health worker’s daily arrival and exit may raise unwanted questions from neighbors or coworkers or create stigma for the patient. In addition, DOT may not always be feasible during adverse weather, natural disasters, or a pandemic [44]. DOT has some substantial disadvantages in clinical practice. First, the implementation requires health, human, and financial resources. DOT also creates a psychological burden for TB patients due to the frequent visits to clinics and the loss of their ability to maintain health privacy [45].

TB treatment can also be self-administered, where the patient receives sufficient medication to perform the complete treatment (in the case of LTBI) or receives medication every three months (in the case of active TB), to be taken without supervision. Eligibility criteria for SA treatment include the capacity of the person with a TB diagnosis to take the medicine alone, their knowledge of the disease, and the absence of a physical or mental disability that would prevent them from taking the medication properly [46].

VDOT has emerged as a potential alternative to DOT in TB treatment and was necessary during the COVID-19 pandemic. In VDOT, the TB treatment can be followed remotely to observe patients taking their medication in front of a computer or smartphone camera, in real time (synchronous VDOT). Another approach is asynchronous VDOT, where patients are asked to send their recorded videos taking their medication via smartphones or computers. Health workers can watch the recordings later, and thus taking the medication and monitoring the video do not need to occur simultaneously. The benefits of VDOT include its potential to monitor treatment adherence remotely, flexibility in terms of time, greater interaction between health workers and patients, and lower cost [45].

The vast majority of international migrants who have entered Brazil illegally currently live in difficult-to-access locations, and are afraid to identify themselves as foreigners when approached. As discussed by Silva et al. (2023) and Maia and Azize (2020) [33,47], the majority of international migrants do not have a permanent address, and when accessing health services, they report false or wrong addresses for fear of being identified.

Many international migrants in the DOT group reported their fear of being exposed by direct observation. Truong, Tanni (2022) and Mangan et al. (2023) [44,45] stated that DOT can generate discomfort in these patients and may not be effective because of migration inside the territory and the lack of valid addresses. The same can also be seen in some cases with VDOT, where some participants may feel uncomfortable recording a video taking their medication, for fear of being tracked by the smartphone app, thus preferring self-administered treatment, which does not entail extensive contact with the health service or health worker. Furthermore, the majority of migrants may not have continuous or any access to the internet.

Immigrants/refugees face vulnerabilities. The main barriers to immigrants’ access to healthcare include cultural differences [48], socioeconomic status, language difficulties, a lack of documentation, an absence of medical history, social isolation, a lack of information about accessing healthcare services, racism, and xenophobia. Additionally, they may encounter restrictions to foreigner care in the local public healthcare system [33]. This can lead to treatment abandonment. Rocha et al. (2020) [49] showed in a study that the main challenges faced by international migrants in accessing healthcare in Brazil were language barriers, cultural issues (conflicts with medicine), scheduling problems (needing to miss work to seek care), and delays in treatment. Other issues noted were episodes of missed appointments and a lack of information, such as obtaining medications [50]. 

Healthcare teams also face challenges in treating immigrants, such as language difficulties and locating patients due to frequent changes in residence or a fear of revealing their address [33]. This may explain the dropout rate in the DOT group. Given this, there is a preference for SA treatment where the patient has autonomy in taking medication and does not need to miss work, for example, to go to the healthcare unit, which inhibits the fear of being located. 

The findings of our study are in line with those of Story et al. (2016) [24], where the author evaluates video- (or virtually) observed therapy (VOT) and presents results that show better participant adherence to VOT compared to DOT. In the study conducted in Manaus-AM, we observed better adherence to treatment in the VDOT group compared to the DOT group, with greater treatment completion and fewer interruptions in the VDOT group. Furthermore, we verified the usability of VDOT in vulnerable and mobile populations, who often do not have a permanent residence to complete treatment. It is understood that not all treatment modalities are suitable for all patients, and this study demonstrates the effectiveness of a new treatment modality for international migrants.

In the sociodemographic aspects of the study, there was a predominance of individuals with brown skin color, corroborating the findings in the literature [51,52,53,54], which demonstrate the vulnerability of these individuals. A literature review conducted by Ferreira et al. (2022) [55] selected 27 articles that reported on the characteristics of TB treatment abandonment. Of these 27 studies, 26 reported a predominance of abandonment among males. The same study showed a significant relationship between race/skin color and treatment abandonment, specifically among those with brown skin color. The Brazilian Society of Tropical Medicine also points out that the black or brown population, living in situations of social vulnerability, is twice as likely to contract TB [56]. Effectively combating LTBI among international migrants lies in an integrated and culturally sensitive approach. Global health authorities must consider and address racial and cultural inequities by ensuring that all migrants have access to quality healthcare. Only through inclusive policies, education, improved access, and collaboration between different entities will it be possible to reduce the prevalence of LTBI and improve the health of these vulnerable populations.

Another stigma related to the negative outcome of tuberculosis treatment is associated with drug or alcohol use. The studies identified that often, the association between treatment and drug use leads patients to a lesser understanding of the importance of regular and complete treatment [55]. It is also important to mention the shortage of the tuberculin skin test for four months, directly impacting the study’s results, since it was not possible to follow all participants to the end of their treatment. 

The limitations of this study include the international migrants’ lack of (continuous) access to the internet, their lack of access to smartphones, and the low specificity of PPD for diagnosing LTBI. However, in Brazil, PPD is recommended by the World Health Organization and the Ministry of Health for detecting LTBI and tracking TB contacts. Therefore, we suggest carrying out future studies with different approaches to screening and detecting LTBI in international migrants, refugees, and stateless persons. A survey carried out in 2019 by the Community Communication Working Group of the R4V platform [57] revealed that 65% of refugees and migrants from Venezuela in Brazil have access to a cell phone, and that 80% access the internet through different devices. To minimize the difficulty of accessing smartphones and the internet, smartphones and internet packages were made available between the study groups.

The study participants’ cultural and social diversity emphasizes the importance of a holistic approach to the implementation of health programs. The cascade of care reveals the complexity of the participants’ trajectory in the health system, indicating the need for strategies to mitigate the specific challenges faced by the migrant population. Despite all the investments to guarantee tuberculosis preventive treatment, there are cultural issues that must be considered and that may have impacted the results. We found that most individuals who dropped out of treatment were indigenous people, thus highlighting the importance of defining a strategy aligned with anthropology, psychology, and human and social sciences. There is consensus that guaranteed access to health services and health education and the supply of culturally appropriate TB services are key components in LTBI screening programs for immigrants [33].

Tuberculosis preventive treatment poses a significant challenge in Brazil, since it is necessary to educate and convince people that they are ill and that they may develop the disease in the future, making preventive treatment necessary. Treatment with the 3HP regimen is an excellent alternative due to its weekly dosage and short duration (12 weeks). However, changing cultural mindsets is a gradual process that does not occur overnight. It is also imperative to encourage physicians, nurses, and community health agents to be proactive in the identification of individuals who have been in contact with someone with active TB. These people may be infected and at risk of developing the disease.

## 5. Conclusions

The results confirmed the efficacy of VDOT in ensuring the adherence to and completion of TB preventive therapy among migrants and refugees, demonstrating that it is the most successful method. This study significantly contributes to the understanding of TB dynamics among international migrants living in Manaus, Amazonas, emphasizing the importance of adaptability and personalization in intervention and treatment strategies for this population.

The results show that relying on different treatment strategies, adapted to the individuals’ needs and risk factors, is a viable and effective way to provide person-centered TB care. Considering the barriers identified here, it is crucial to develop future studies with new approaches to the prevention and treatment of TB among migrant populations.

Tuberculosis remains an essential area of research compared to other infectious diseases due to its persistent prevalence, complexity in transmission, and significant impact on global public health. TB presents unique challenges, such as the need for prolonged treatment and the emergence of drug-resistant strains, which require innovative and specific interventions. Therefore, it is essential to continue investing in TB research to develop more effective control strategies, especially in vulnerable populations such as international migrants.

## Figures and Tables

**Table 1:** Main characteristics according to treatment modality of short-term LTBI with the 3HP regimen among international migrants, Manaus, Amazonas, Brazil (2022–2023).

	SA (*n* = 31; 100%)	DOT (*n* = 26; 100%)	VDOT (*n* = 31; 100%)
Gender			
Male	18 (58.1%)	15 (57.5%)	16 (51.6%)
Female	10 (32.2%)	11 (42.3%)	15 (48.4%)
Other	3 (9.7%)	0 (0%)	0 (0%)
Age group			
18 to 39 years old	17 (54.9%)	17 (65.4%)	17 (54.9%)
40 to 59 years old	13 (41.9%)	9 (34.6%)	10 (32.2%)
60 years or older	01 (3.2%)	0 (0%)	04 (12.9%)
Race/Color/Ethnicity			
Asian descendant	2 (6.5%)	0 (0%)	1 (3.2%)
White	2 (6.5%)	1 (3.8%)	0 (0%)
Indigenous	8 (25.8%)	9 (34.6%)	9 (29.0%)
Brown	15 (48.4%)	13 (50.0%)	13 (41.9%)
Black	4 (12.9%)	3 (11.5%)	8 (25.8%)
Marital status			
Married	5 (16.1%)	10 (38.5%)	9 (29.0%)
Separated/Divorced	0 (0%)	0 (0%)	1 (3.2%)
Single	26 (83.9%)	15 (57.7%)	21 (67.7%)
Widow(er)	0 (0%)	1 (3.8%)	0 (0%)
Education			
Higher education	3 (9.7%)	2 (7.7%)	3 (9.7%)
Between 5 and 8 years of study	9 (29%)	12 (46.2%)	11 (35.5%)
Between 9 and 11 years of study	12 (38.7%)	5 (19.2%)	10 (32.3%)
Did not study	2 (6.5%)	2 (7.7%)	1 (3.2%)
Did not answer	5 (16.1%)	5 (19.2%)	6 (19.3%)
Treatment outcomes			
Incomplete treatment/Dropout/Moved abroad	5 (16.1%)	6 (23.1%)	6 (19.4%)
Complete treatment/Adherence	26 (83.8%)	20 (77%)	25 (80.6%)

**Table 2:** Main characteristics according to the treatment modality of short-term LTBI with the 3HP regimen among international migrants with incomplete treatment or noncompliance and complete treatment or adherence, Manaus, Amazonas, Brazil (2022–2023).

	SA		DOT		VDOT	
	Incomplete (*n* = 5)	Complete (*n* = 26)	Incomplete (*n* = 6)	Complete (*n* = 20)	Incomplete (*n* = 6)	Complete (*n* = 25)
Gender						
Male	3 (60%)	15 (57.7%)	5 (83.3%)	10 (50%)	3 (50%)	13 (52%)
Female	2 (40%)	8 (30.8%)	1 (16.7%)	10 (50%)	3 (50%)	12 (48%)
Other	0 (0%)	3 (11.5%)	0 (0%)	0 (0%)	0 (0%)	0 (0%)
Age						
18 to 39 years old	3 (60%)	14 (53.9%)	6 (100%)	11 (55%)	4 (66.7%)	13 (52%)
40 to 59 years old	2 (40%)	11 (42.3%)	0 (0%)	9 (45%)	2 (33.3%)	8 (32%)
60 years or older	0 (0%)	1 (3.8%)	0 (0%)	0 (0%)	0 (0%)	4 (16%)
Race/Color/Ethnicity						
Asian descendant	0 (0%)	2 (7.7%)	0 (0%)	0 (0%)	1 (20%)	0 (0%)
White	0 (0%)	2 (7.7%)	0 (0%)	1 (5%)	0 (0%)	0 (0%)
Indigenous	3 (60%)	5 (19.2%)	4 (66.7%)	5 (25%)	3 (60%)	6 (24%)
Brown	2 (40%)	13 (50%)	2 (33.3%)	11 (55%)	1 (20%)	12 (48%)
Black	0 (0%)	4 (15.4%)	0 (0%)	3 (15%)	1 (0%)	7 (28%)
Marital status						
Married	1 (20%)	4 (15.4%)	2 (33.3%)	8 (40%)	1 (16.7%)	8 (32%)
Separated/ Divorced	0 (0%)	0 (0%)	0 (0%)	0 (0%)	0 (0%)	1 (4%)
Widow(er)	0 (0%)	0 (0%)	0 (0%)	1 (5%)	0 (0%)	0 (0%)
Single	4 (80%)	22 (84.6%)	4 (66.7%)	11 (55%)	5 (83.3%)	16 (64%)
Education						
Higher education	0 (0%)	3 (11.5%)	0 (0%)	2 (10%)	0 (0%)	3 (12%)
Between 5 and 8 years of study	3 (60%)	6 (23.1%)	3 (50%)	9 (45%)	0 (0%)	9 (36%)
Between 9 and 11 years of study	2 (40%)	10 (38.5%)	1 (16.7%)	4 (20%)	3 (50%)	7 (28%)
Did not study	0 (0%)	2 (7.7%)	0 (0%)	2 (10%)	0 (0%)	1 (4%)
Did not answer	0 (0%)	5 (19.2%)	2 (33.7%)	3 (15%)	3 (50%)	5 (20%)

**Table 3:** Efficacy of treatment modalities in adherence † to short-course treatment (3HP) among international migrants and refugees, Manaus, Amazonas, Brazil.

Treatment Modalities	Adherence		
	OR_Crude_ (95%CI)	OR_Adjusted_ * (95%CI)	(*p*-Value)
SA	4.86 (1.18–4.86)	9.93 (1.96–50.30)	0.006 **
DOT	0.69 (0.45–2.04)	0.59 (0.14–2.38)	0.455
VDOT	0.32 (0.31–0.32)	0.26 (0.7–0.94)	0.04 **

† Used the variable ‘Complete treatment’, classifying 0 for ‘yes’ and 1 for ‘no’. * Odds ratio adjusted for gender, age, race/color, and marital status. ** *p* value < 0.05. −2 Log likelihood: 63.6; Cox and Snell R Square: 0.32; Nagelkerke R Square: 0.43.

**Table 4:** Description of those who dropped out of treatment for LTBI, among international migrants and refugees, Manaus, Amazonas, Brazil.

Gender	N (%)
Male	12 (70.59%)
Female	5 (29.42%)
**Race/Color/Ethnicity**	
Indigenous	8 (47.06%)
Brown	6 (35.29%)
Black	1 (5.88%)
White	2 (11.76%)
**Marital status**	
Single	12 (70.59%)
Married	5 (29.41%)

## Data Availability

The data presented in this study are available from the corresponding author on reasonable request due to privacy and identification the participantes.
